# Progress and persistence of diseases of high consequence to livestock in the United States

**DOI:** 10.1016/j.onehlt.2024.100865

**Published:** 2024-07-29

**Authors:** Mark R. Ackermann, John P. Bannantine

**Affiliations:** US Department of Agriculture-Agricultural Research Service, National Animal Disease Center, Ames, IA, USA

**Keywords:** Animal disease, Animal health, One health, USDA, Zoonotic diseases

## Abstract

The USDA/ARS-National Disease Center (NADC) will celebrate its 65th anniversary of existence in November 2026. NADC continues as one of the world's premier animal health research centers conducting basic and applied research on endemic diseases with economic impact on U.S. livestock and wildlife. This research center also supports a program studying important food safety pathogens such as *Salmonella*, *E. coli* and *Campylobacter*. NADC has contributed significantly to the elimination of a few diseases, notably hog cholera and milk fever, and made progress in reducing the impact of many other animal diseases through vaccines, therapies and managerial recommendations. Despite nearly 65 years of targeted research on these diseases and much progress, some of these continue to persist. The reasons for such persistence varies for each disease condition and they are often multifactorial involving host susceptibility, virulence and even environmental conditions. Individually and in aggregate, these disease conditions have a massive economic impact and can be devasting to animal producers, owners and individuals that become infected through zoonotic disease agents such as tuberculosis, leptospirosis and avian influenza. They also diminish the health, well-being and welfare of affected animals, which directly affects the food supply. The NADC is using all available technologies including genomic, biochemical, reverse genetics, and vaccine trials in the target host to combat these significant diseases. We review the progress and reasons for persistence of selected diseases and food safety pathogens as well as the progress and potential outcomes should research and programmatic plans to eliminate these disease conditions cease.

## 1 Introduction

The National Animal Disease Center (NADC) is a 523 -acre stand-alone research facility located in central Iowa with the long-standing mission of reducing and eradicating high consequence animal diseases endemic to the United States. Exotic animal diseases are the research focus of a newly constructed facility in Manhattan Kansas – The National Bio and Agro-Defense Facility (NBAF). Both facilities are owned by the U.S. Department of Agriculture (USDA) and within the Agricultural Research Service, which is the research agency of the USDA. Another USDA agency, the Animal Health and Plant Inspection Service (AHPIS) and the Department of Homeland Security are also housed at these locations (NADC and NBAF, respectively). Although NBAF's legacy is just starting, NADC has been conducting research for >60 years. This length of time has provided an opportunity to assess the high impact research conducted at NADC and shows that several diseases have been eradicated or their impact significantly reduced [1]. NADC's facilities were updated/modernized 15 years ago and includes a biosafety level-3 large animal high containment facility with flexible room designs and animal pen layouts that enable bison, cattle, deer, elk, swine and other animals to be studied in a highly controlled environment that is severe weather resistant and bio-secure.

Animal health research conducted at NADC can be divided into diseases affecting humans and animals as well as diseases affecting only animals. The zoonotic diseases have obvious One Health implications, yet NADC also investigates a multitude of endemic disease conditions that are difficult to handle and eliminate (Table 1). Work over the last 65 years at NADC has resulted in great progress in reducing the impact of these disease conditions on animal health, the economy, food safety and zoonotic disease outbreaks (Table 2), but the persistence of these disease conditions means that they are still problematic causing suffering, pain, and interspecies spread by viral and bacterial pathogens including the potential spread to humans. The dynamic contrast of progress in reducing the incidence and/or impact of these disease conditions with dogged persistence, begs the question as to what factors contribute to the persistence. For many of these, the reason(s) for persistence are multifactorial and complex.

**Table 1:** Conditions persistent to key livestock diseases endemic to the United States, progress in combating these conditions, and current efforts to understand and mitigate these disease conditions at the USDA/ARS-National Animal Disease Center (NADC).

Disease condition	Reason(s) for persistence (selected)	NADC accomplishments/actions and references
Bovine tuberculosis	Lack of support to test and eradicate, no fully effective vaccine, and resistance of eradication by slaughter	Characterized bovine tuberculosis disease progression in cattle, deer, elk [[74], [75], [76]]. Vaccination of wild deer with BCG bait underway in Michigan
Brucellosis	Lack of vaccine effective for elk, resistance of eradication by slaughter	Characterized brucellosis in cattle, bison and key to RB51 vaccine development [18,77]. Identified poor vaccine response in elk and developing new generations of vaccines [20,78].
Transmissible Spongiform encephalopathies (Chronic wasting disease, Bovine spongiform encephalopathy, Scrapie)	Persistence of organism in environment and progressive spread	Discovered interspecies transmission [41,42], retinal accumulation [79], prion variability [44], novel diagnostic tests [79], biochemical detection and biochemical properties that contribute to disease [41].
Leptospirosis	Rodent/animal reservoirs and survival in water	Discovered new strains [32], new media for growth and advancing toward new vaccines with CRISPR technology [37,40].
Digital Dermatitis	Complex etiology and environmental survival	Created a model in ruminants used to characterized disease progression and demonstrating Biocide treatment efficacy [48,49].
Contagious Bovine Pleuropneumonia (CBPP)	In Africa, no fully effective vaccine or therapy and lack of political cohesion to eradicate by slaughter	Creation of a new project in collaboration with colleagues in Kenya and University of Connecticut, created a vaccine
Influenza in swine	Reassortment/recombination, passage from human to swine (and swine to human)	Discovered virulence of various strains of influenza in swine, efficacy of vaccines, sequence assessment for prediction of spread/virulence and interspecies transmission [80]
Highly Pathogenic Avian Influenza (HPAI) in dairy cattle	Reassortment/recombination and spread by birds, between cattle and susceptibility by numerous mammals	First in the world to elucidate sequence data of HPAI strains and first in the world to infect heifers and lactating cows experimentally. Plans to create an experimental model used to clarify pathogenesis and use for vaccine studies.
*Mycobacterium avium paratuberculosis*	This organism persists in the environment and spreads to young animals housed in the same area	Identification of diagnostic antigens and vaccines that reduce pathogen shedding in dairy cows [73,81]. Sequenced the genomes of cattle and sheep strains [[82], [83], [84]].
Food Pathogens, *Salmonella* spp.	Housing/management practices, susceptibility of various host species and lack of universal vaccine	Discovered molecular mechanisms for colonization/virulence, created and tested vaccines included DIVA vaccines. Discovered immune responses to colonization and potential immune mechanisms of resistance. Discovered types of mechanisms of antimicrobial resistance, microbiome influences and nutritional substances to alter the microbiome/immunity [70,85,86].
Food Pathogens, C*ampylobacter* spp.	Housing/management practices and lack of universal vaccine	Discovered molecular mechanisms for colonization/virulence. Discovered immune responses to colonization and potential immune mechanisms of resistance. Discovered types of mechanisms of antimicrobial resistance, microbiome influences and nutritional substances to alter the microbiome/immunity.
Food Pathogens, *E. coli O157:H7*	Persistence in colonization of cattle	Discovered key site of colonization, the rectoanal junction and created models of microbial adherence and with this discovered key molecular mechanisms of colonization [87].
Mastitis	Housing/management practices and susceptibility of dairy cows due to modern genetics	Developed experimental models of mastitis and immune genes that contribute to mastitis resistance by comparing genes of cattle with modern genetics to historical (1964) genotypes [68].

**Viral infections of swine**		
Porcine reproductive and respiratory syndrome virus	Housing/management practices and immune responsiveness	Pathogenesis characterization of Asian and U.S. virulence strains [88], innate immune responses, vaccine development with attenuated and recombinant strains.
Swine enteric coronaviruses; porcine epidemic diarrhea virus (PEDV), betacoronavirus	Housing/management practices and immune responsiveness	Characterized interferon responses and therapeutic intervention against porcine epidemic diarrhea virus A (PEDV); Determined immune response drivers against neurotropic betacoronavirus porcine hemagglutinating encephalomyelitis virus (PHEV) including interferon and chemokine responses involved in antiviral regulation
Senecavirus A	Housing/management practices and immune responsiveness	Demonstrated cattle are likely not susceptible to infection with Senecavirus A (SVA); tested inactivated vaccine [89].
Pseudorabies virus	Housing/management practices and immune responsiveness	Determined gene sets associated with pseudorabies virus infection in feral swine
Circovirus	Housing/management practices and immune responsiveness	Strain virulence and vaccine development
Atypical porcine pestivirus (APPV)	Housing/management practices and immune responsiveness	Reproduced congenital tremors and clinical features of atypical porcine pestivirus (APPV) in piglets and demonstrated longitudinal persisitence [90].
Swine influenza infection	Housing/management practices and immune responsiveness	Determined evolution of H1N1, H1N2, and H3N2 whole genome sequence based on the frequency of reassortment among the segments; discovered a live vaccine expressing an IgA inducing protein protects pigs; assessed interspecies transmission from pigs to ferrets and antigenically drifted contemporary H3N2 risk for human transmission; discovered Mucin 4 is a cellular biomarker of necrotizing bronchiolitis [91]; assessed role of transmembrane protease serine S1 member 2 protease in reducing disease severity [92].
SARS-CoV-2	Persistence in white-tailed deer	First in the world to discover that white-tailed deer are highly susceptible to SARS-CoV-2 infection [93,94] and assessed responses in numerous other species, including mink and elk [95].

**Swine bacterial pneumonia**		
*Streptococcus suis*	Housing/management practices and immune responsiveness	Virulence and antimicrobial gene expression [96]; colonization and virulence in vivo; determine effects of colonization using co-infection model [97].
*Streptococcus equi subspecies* *zooepidemicus*	Housing/management practices and immune responsiveness	Virulence in vivo and response to ceftiofur therapy; discovered a lack of vaccine protection with a bacterin [98].
*Pasteurella multocida/Bordetella bronchiseptic*	Housing/management practices and immune responsiveness	Colonization and virulence and impact on nasal microbiota [99], effects of co-infection with *S. suis* and virulence/biofilm gene characterization [100].
*Glaesserella parasuis*	Housing/management practices and immune responsiveness	Discovered effect of polysaccharide capsule on virulence with consideration of vaccination and antimicrobial resistance [101].

**Bovine viral pneumonia**		
Bovine viral diarrhea virus	Housing/management practices and immune responsiveness	Discovered that a biotherapeutic protein can substantially neutralize BVDV in a concentration-dependent manner. Determining the role of tRNA fragments on immune responses and virus neutralization titers among BVDV isolates [102].
Bovine herpes virus-1	Housing/management practices and immune responsiveness	Determined the effect of maternal antibody on calf vaccination and assessing role of BHV-1 co-infection with *Mycoplasma bovis*.
Bovine respiratory syncytial virus	Housing/management practices and immune responsiveness	Developed and characterized a calf model; tested intranasal recombinant NDV-RSV F opt vaccine [103].

**Bovine bacterial pneumonia**		
*Mannheimia haemolytica*	Housing/management practices and immune responsiveness	Differential identification of *Mannheimia haemolytica* genotypes 1 and 2 and transcription profiles [104]; creation and use of a *M. haemolytica* vaccination platform [53].
*Pasteurella multocida*	Housing/management practices and immune responsiveness	Purified lipopolysaccharide (LPS) from both *M. haemolytica* and *P. multocida* wildtypes and sialic acid mutants and discovered their effects on peripheral blood mononuclear cell responses [105].
*Histophilus somni*	Housing/management practices and immune responsiveness	Demonstrated antimicrobial activity of four bovine NK-lysin-derived peptides against bovine *H. somni* isolates [106]
*Mycoplasma bovis*	Housing/management practices and immune responsiveness	Demonstrated a vaccine with *M. haemolytica* expressing (and secreting) *Mycoplasma bovis* antigens protects cattle from *M. bovis* lung challenge [53]; determining serum antibody (IgG) role in bison for recovery of *M. bovis* infection.
*Mycoplasma mycoides mycoides (Contagious Bovine Pleuropneumonia)*	International concern in cattle exacerbated by lack of an effective vaccine and managerial policies	Established projects with Kenyan laboratories and set up NADC capability. Created an *M. mycoides mycoides* vaccine in the *M. haemolytica* platform and assessed responses by vaccinated cattle.

**Table 2:** Selected examples of rapid response research projects for newly arising/emerging disease conditions at the USDA/ARS-National Animal Disease Center (NADC) since 1986.

Year [citation]	Disease Condition	NADC Response
1986	Bovine Spongiform Encephalopathy	Established a transmissible spongiform encephalopathy project that now includes Scrapie, Chronic wasting disease and Mink Encephalopathy
1986 and 2017 [88]	Porcine Reproductive and Respiratory Disease Virus (PRRSV)	Pathogenesis studies to determine strain virulence, secondary infections, and vaccine development
1995 through the present day [107,108]	Tuberculosis in white-tailed deer (WTD)	Established a project and ability to work with WTD in experiments to characterize disease development and develop vaccine strategies in trial today
1998	Epizootic Hemorrhagic Disease in WTD	Assessed virulence of virus and established pathogenesis
1990's and 2004 [109]	HoBi-like pestivirus	Established experiment model
2006 [110,111]	Highly pathogenic Asian strain of PRRSV	Established virulence of strains in porcine model and contributed to vaccine development
2013 [112]	Asian Porcine Endemic Diarrhea virus (PEDV)	Established virulence in porcine model and project for continued study of PEDV
2014	Second strain of Asian PEDV	Established virulence in porcine model and project for continued study of PEDV
2014 [113]	Porcine delta coronavirus	Established virulence in porcine model and project for continued study of delta coronavirus
2015 [114,115]	Senecavirus A	Established model to characterize disease severity and transmission and for continued study of Senecavirus A
2015	H5N8, H5N2, H5N1; canine H3N2	Assessed virulence and transmission in swine
2020 [93,94]	SARS-CoV-2	First to discover SARS-CoV-2 readily infects deer which lead to discovery of widespread distribution in wild WTD in the US.
2024	Highly Pathogenic Avian Influenza (HPAI) infection of Dairy Cows	Sequence analysis demonstrating evolution of the condition and first in the world to infect heifers and lactating dairy cows experimentally

### 1.1 Diseases with a research focus at NADC

We present a high-level view of selected diseases studied at NADC with the goal of showing the diversity and impact that research has on combating animal diseases, many of which are very difficult to work with due to persistence factors. Bovine tuberculosis, Brucellosis, prion diseases, leptospirosis, avian influenza and food safety pathogens are among the diseases with One Health implications. Other diseases with high consequences for animal health include Contagious Bovine Pleuropneumonia (CBPP) and digital dermatitis, a primary cause of lameness in cattle, elk and other ruminants.

### 1.2 Bovine tuberculosis

Bovine tuberculosis (bTB) is considered a primary example of a One Health disease affecting humans, livestock and wildlife. The primary host for *Mycobacterium bovis* is cattle, but it can also cause disease in a range of wildlife species including badgers, possums, deer and ferrets [2]. An eradication program for bTB caused by *M. bovis* began in 1917. At that time, 5% of the U.S. cattle were infected and there were 25,000–50,000 human deaths from *M. bovis* each year. Although animal workers are known to acquire the disease by inhaling cough droplets from infected cattle [3], human infections were reduced significantly with the discovery of milk pasteurization in the 1940s. The percentage of bTB disease dropped from 9% in 1938 to 1.5%–2.0% by 1952 in Amsterdam [4]. Since that time, there has also been a massive reduction in the incidence of bTB in cattle, wildlife and U.S. citizens. However, there is a wildlife reservoir that harbors *M. bovis* in most areas of the globe where bTB is endemic [5]. Even today, *M. bovis* persists in the White-Tailed Deer (WTD) population of four counties in Michigan. These are Alcona, Alpena, Montmorency, Oscoda counties where there was a 4.9% WTD carrier rate in 1994 which decreased to <1% in 2014 [6]. Even with less than a 1% carrier rate, *M. bovis* can persist in the endemic area, perhaps partly due to the low infectious dose of as little as 1 colony forming unit [7]. In fact, >99% of bTB positive WTD in the U.S. are from endemic areas while only 0.008% are from outside these areas. One way to potentially eliminate deer carrying *M. bovis* in this area could include slaughter of deer, which was a successful strategy to eliminate *M. bovis* in WTD in Northern Minnesota [8]. However, in Michigan, slaughter has not occurred due to a variety of social and logistic factors [9,10]. Additional factors that have maintained bTB in this area include antler point restrictions which force hunting of younger deer with smaller antlers and allows older deer to survive, focal and organized efforts to allow baiting and feeding deer (carrots, sugar beets, apples) which allows aggregation of deer [11], fewer hunting licenses sold (less interest in hunting) with 872,000 sold in 1995 and 582,000 in 2019, changes in attitude/understanding of inhabitants in these counties (living with animals preferably over domination of the land and wildlife), and distrust of natural resource managers by some landowners and some hunters [9]. Yet another concern for bovine tuberculosis in the United States are pockets of feral pigs in Molokai, Hawaii that carry *M. bovis* [12]*.*

Despite these broad issues that require multiple regulatory agencies to agree on control strategies, there remain staunch research efforts to reduce *M. bovis* in these endemic counties. NADC scientists initially identified that at least one route of transmission was through contaminated feeding areas for deer and other wildlife. They found that *M. bovis* could persist on a variety of feedstuffs for up to 112 days [11]. This environmental persistence has led to spillover infections from deer to cattle, and even humans, in those counties [13]. Recently whole genome sequencing (WGS) of *M. bovis* isolates from Michigan has been used to determine transmission models in deer, cattle and elk populations [14]. WGS data suggest the high-risk periods of transmission are around the time of calving and that herd-to-herd transmission is not observed [10]. In 2024, NADC scientists and collaborators began a vaccination study using BCG-vaccine delivered in edible alginate vaccination units to wild deer (unpublished data). BCG is used because in experimental studies, BCG vaccination reduces *M. bovis* disease severity in WTD [15]. Also, oral vaccination with BCG in free-ranging cattle in Australia prevented infection 67% and a very recent study in Ethiopia using BCG vaccination in a mechanistic transmission model reduced *M. bovis* infection by 24% and could potentially reduce transmission by 89% over time [16].

### 1.3 Brucellosis

Like bovine tuberculosis, Brucellosis is another zoonotic disease with one health implications that also pose a major threat to public health. In the United States, currently a major concern for *Brucella abortus* infection occurs in bison and elk in the Greater Yellowstone Area (GYA). Brucellosis is already endemic in GYA elk, and the disease occasionally spills over into cattle or bison. Roughly 60% of female bison in GYA have titers to *B. abortus* and about 10–15% of female bison can transmit the organism. Although these bison generally stay within the GYA, they come into contact with Rocky Mountain elk which travel in and out of the GYA. The elk also travel to designated feeding areas where they can aggregate and thus pick up the bacterial agent from bison and/or spread it among the elk and, further, to cattle or other species in the vicinity [17]. The vaccine used for cattle, RB51, reduces *B. abortus* colonization and abortion in cattle and has efficacy in bison, depending on delivery method [18]; however, this vaccine does not protect elk against *B. abortus* infection and abortion [19,20]. Thus, a vaccine for elk is needed and investigators at the NADC are characterizing the elk immune system [21] and genome [22] in order to create and test effective vaccines. RB51 is a live *Brucella* vaccine strain that has been tested in a variety of ways including duration, method of delivery, host genotype, synergism with other vaccines and immune parameters [[23], [24], [25], [26]].

Scientists at NADC are also focusing on the GYA problem through a variety of pathogenic and vaccine approaches. A recent study traced individual bacterial cells, each with its own transposon signature, through cattle to determine how long it takes to get from the site of infection to local lymph nodes as well as where bottlenecks to colonization occur [27]. Beyond the GYA problem of *B. abortus* in cattle, bison and elk, other species of *Brucella* affect animals including, *B. melitensis* in small ruminants and cattle, *B. canis* in dogs throughout the world, including the U.S., and finally *B. suis* in wild swine in the U.S. [28], Europe, Australia and other countries and in wild boar, European brown hares and wild red foxes. Brucellosis is prevalent in Mediterranean Europe, Peru, Mexico, Africa, Near East countries, Central Asia, India and Italy and less prevalent in Canada, Australia, Cyprus, Norway, Finland, the Netherlands, Denmark, Sweden, New Zealand and United Kingdom [29]. There is a global incidence of 2.1 million humans with Brucellosis and Africa and Asia sustain the most risk for new cases [30].

### 1.4 Leptospirosis

Staying on the One Health theme of bacterial infections, leptospirosis is another zoonic disease, but this one is transmitted through contaminated water. Outbreaks occur worldwide with a recent one in Tanzania that resulted in 3 mortalities and 20 severely sick [31]. Numerous host species can be infected by various strains and serovars of *Leptospira* sp. Infected animals can carry this agent for long periods of time in their renal tubules and spread the pathogen through excreted urine [32,33]. Rodents are the most significant reservoir host globally and outbreaks can be associated with flooding and increases in rodent populations. Humans can be infected by urine contact on mucosal surfaces or skin abrasions [34]. Leptospirosis can be prevented with diagnostic surveillance, control of reservoir hosts, treatment and vaccination; however, all these countermeasures are not fully effective. Studies by NADC investigators have shown that *Leptospira* sp. occur in 7% of Iowa beef cattle and 14% of California dairy cows. *Leptospira* sp. were also detected in 1.5% of bovine semen [35]. In addition, new serovars (Tarassovi) have been discovered which are not formulated in current vaccines [36]. And recent work has shown that virulent strains of leptospira can be detected in over 30% of rats in the cities of Boston and New York.

NADC investigators created a new growth media to isolate Leptospira and this has allowed comprehensive in vitro culturing at 29 °C and 37 °C for these fastidious organisms [37]. Application of this media formulation led to a better level of detection of viable organisms in samples and identification of new species [33,38]. Work is ongoing to develop DIVA vaccines and live attenuated vaccines using knockout mutations introduced in Leptospira through CRISPR technologies [39] that may provide cross protection against other serovars and species. In one example, expression of a predominant membrane protein, LipL32 was knocked down using CRISPRi technology to evaluate its role in virulence. They found the LipL32 knockdown appeared to promote disease [40]. Conversely, a double knockdown of LigA and B, which binds host ligands, could still persist in the hamsters but could not cause disease [40].

### 1.5 Transmissible spongiform encephalopathies and chronic wasting disease

Transmissible Spongiform Encephalopathies are neurodegenerative diseases caused by altered forms of normal cellular prion proteins (encoded by the host PRNP gene) through an incompletely characterized post-translational process. This results in a cascading event whereby altered prion proteins (Scrapie forms) aggregate and damage neurons and other cells of the central nervous system. Animal TSE's include bovine spongiform encephalopathy (BSE) in cattle, chronic wasting disease (CWD) in certain types of deer, including WTD and elk, Scrape in sheep, transmissible mink encephalopathy (TME) in mink, and camelid prion disease (CPD) in camelids. In the US, there have been only four incidents of BSE identified and the incidence of Scrapie has decreased dramatically over the last decade, particularly in the last five years. However, CWD is spreading rapidly in WTD and elk, with hotspots in defined areas of the US. Overall, it is present in low abundance in nearly all 50 U.S. states and Canada.

The reason(s) for CWD spread are not fully understood and likely multifactorial. But a very significant issue is that the abnormal prion protein is highly resistant to proteolytic degradation and can exist for years to decades in many conditions or environments. Transmission among deer and elk can occur through saliva, feces, blood, and they are disseminated by carnivores such as raccoons or scavengers such as birds. Spread can also occur by hauling of carcasses by hunters and/or prey; feeders, waters, bait; and other reservoirs such as other susceptible species (reindeer, sika, mule deer, muntjack deer) and those with unknown susceptibility (e.g., exotic ungulates and potentially other ruminants not yet tested such as black-tailed deer, caribou). Other research gaps for CWD include: the dose needed to establish infection and interspecies transmission among carnivores.

Researchers at NADC have examined interspecies transmission of the scrapie and CWD agents [41,42] and PRNP genotypes of sheep that make them more resistant to scrapie [43; 44]. They discovered that the misfolded scrapie prion protein from sheep, which is 100% infectious in deer, changes to resemble another common scrapie prion protein after passage through deer [41]. They also showed that sheep were susceptible to the scrapie agent obtained from deer just as deer are susceptible to sheep scrapie [42]. Regarding host resistant genotypes, researchers discovered sheep that are homozygous for lysine at position 171 of the prion protein were more resistant to infection with the scrapie agent [43]. Finally, researchers have been able to distinguish scrapie strains based on phenotypic profiles including incubation time to clinical disease, susceptibility, and transmissibility [44].

### 1.6 Digital dermatitis

Lameness in dairy cows is the second major health concern for the dairy industry behind mastitis, another disease studied at NADC [45,46]. Digital dermatitis is the leading cause of lameness in dairy operations and beef cattle feedlots costing over $300/incident due to production loss, treatment, prevention and early culling [47]. Digital dermatitis is a polymicrobial infection of the skin at the hoof junction with Treponeme organisms present at the leading edge of such lesions. Digital dermatitis was first reported in 1974 and has slowly spread among dairy and beef cattle as well as sheep, goats and wildlife (Mediterranean Buffalo, European Bison, wild elk). The polymicrobial nature of this disease condition and continual spread in cattle, sheep, goats and insidious spread among wildlife are indications that this disease will persist. At NADC, investigators have tested a degradable hydrazone biocide that reduces disease severity with several 90-second treatments in a footbath (unpublished). These researchers have also characterized the microbiome present in digital dermatitis lesions via 16S rRNA sequencing [48], developed a sheep infection model [49], and defined T lymphocyte populations present in diseased cattle [50].

### 1.7 Contagious bovine pleuropneumonia (CBPP)

Until a recent update to OIE disease notifications, CBPP was the only OIE A listed disease caused by a bacterial pathogen. *Mycoplasma mycoides* subspecies *mycoides* is the etiologic agent of CBPP and was eradicated by a policy of compulsory slaughter in North America, most of Europe (except the Iberian Peninsula and parts of the Balkans), and Australia in the late 1800's. However, CBPP is still present in sub-Saharan Africa in cattle and also Asian buffalo, captive bison, and yak [51]. In African countries with CBPP it can cause up to 50% mortality in herds infected for the first time. Eradication is difficult in Africa because some infected animals become carriers and can cross borders in nomadic regions [52]. Also, vaccines are not fully effective, antibiotic use can be sporadic and some antibiotics brought into some parts of Africa are not well manufactured and vetted or stored properly. Vaccine development is hampered by the need for an experimental model of CBPP for testing in the laboratory as well as the investment to create new vaccines. Scientists at the NADC have a new project in CBPP and have recently created a vaccine based on a modified *Mannheimia haemolytica* vector platform for expressing proteins derived from the closely related strain *Mycoplasma bovis* [53]. These studies, along with others in the planning stages, will provide the foundation for developing CBPP vaccines.

### 1.8 Influenza in swine and dairy cattle

Influenza viruses undergo reassortments and shifts of their genes, particularly in genes encoding the capsid proteins hemagglutinin (H) and neuraminidase (N), that can change its virulence within host species. Such reassortments can also allow the virus to jump to another species such as from human to swine. When this occurs, H genes reassort frequently resulting in rapid phylogenetic changes after defined lineages enter swine. Such strong genetic drifts in these variable genes often result in pandemics. The Influenza A virus responsible for the 2009 H1N1 pandemic, pdm09, has passed from humans to swine over 400 times since 2009 [54,55], yet only a few of these viruses become established in swine due to an increased affinity for the lower respiratory tract and alveolar macrophages [56]. Subsequent circulation in swine has resulted in pdm09 variants that have jumped from swine back to human and these swine-circulating variants are often poor matches for human seasonal flu vaccines. Thus, influenza viruses have a natural ability to change their genes and then can spread to other species and human strains, once in swine, can change and go back to humans with little human protection from seasonal vaccination.

An NADC scientist and collaborators have developed molecular epidemiological and bioinformatic tools to enable surveillance and tracking of new influenza strain variants across the globe. One of these tools is the influenza research database, which is freely accessible online and includes a suite of visualization and analysis workflows [57]. Among the bioinformatic software developed for influenza genetic diversity and surveillance, PARNAS can identify virus taxa that optimally represent the phylogenetic diversity [58] along with octoFLU [59] and ISU FLUture, a database of influenza virus cases submitted to the Iowa State University veterinary diagnostic laboratory [60].

While poultry, swine, and humans are all well-known hosts for these viruses, the H5N1 strain of Highly Pathogenic Avian Influenza (HPAI) was determined by USDA-APHIS and NADC investigators to occur by a single bird-to-cow transmission event in December 2023 [61,62]. This event was documented through genomic epidemiological methods and made national news outlets since it had never been observed previously. The spillover into cattle was preceded by reassortment events not involving the H and N genes, but rather the PB2 and NP genes [62]. Once in cattle, this viral genotype stabilized and spread among herd mates and infected mammary glands resulting in decreased milk production and changes in rumination. High levels of virus can be present in these infected cows and cats on some farms with HPAI infections died from influenza encephalitis. In response to HPAI infections in this new host, NADC investigators are currently developing a model of infection with H5N1 in dairy cows. Also, the USDA has evidence that the milk supply is safe despite these new HPAI infections.

## 2 Viral and bacterial pneumonia of swine and cattle, food safety pathogens, mastitis, and others

Despite decades of managerial strategies and development of antibiotics and vaccines, cattle, swine, and poultry farms still struggle with pneumonia, diarrhea and contamination of food products with bacteria pathogenic to humans. There have been vast improvements in management in terms of housing and ventilation, although, the congregation/aggregation of animals in the same pens/areas allows passage of respiratory organisms between animals breathing the same airspace and pens can also enable exposure to fecal/urinary waste on floors. This results in a constant exchange of microbiomes between pen mates. Air flow should be managed in many types of housing facilities and slotted flooring and litter types should be used to reduce accumulation of feces and urine. However, in reality, some livestock producer managers and facilities are better than others. In the wild, humans and animals do their best to separate themselves from fecal/urine waste yet some of our animal managerial systems do not have built-in protocols for immediate removal of feces and urine. Small, back-yard herds and flocks can address the density somewhat, but many of these still have animals in the same locations but do not invest in ventilation systems and specialized flooring. It is unwise to depend solely on vaccines and antibiotics to keep animals healthy when there can be subpar housing conditions that are readily fixable.

Swine farms have improved their biosecurity over many decades largely in response to the spread of pathogens that occur when housing a high density of sows especially during farrowing and pigs in nursery, grower/finishing units. In the 1980's there were efforts toward minimal disease and specific pathogen free farms and thereafter there has been biosecurity measures implemented to: introduce replacements and use of semen, control entry and exit of vehicles and people, transport of animals, spacing of farms/units, feed and water [63]. At the same time, there have been advances in ventilation, temperature and humidity regulation, and waste disposal. The high density rearing of pigs decreases the environmental footprint of raising massive numbers of animals, yet along with this solution there are issues related to stress, welfare and public concern [64].

Poultry farms/units have increasingly enhanced their biosecurity measures over the last decade due to the pressures of food pathogens and avian influenza. Like swine farms, many poultry facilities implement biosecurity measures to reduce pathogens entering facilities from infected people and introduction of newly purchased animals. This helps to reduce infections, but pathogens such as *Salmonella* spp. and *Campylobacter* spp. remain issues for both conventional and organic poultry farms. These organisms are monitored by quantitative microbial risk assessment (QMRA) by the food industry and some QMRA attempt to model these pathogens on conventional farms from farm-to-fork or retail-to-consumption [65]. Organic, pastured and free-range poultry farms and their antibiotic-free nature is attractive to many consumers; however, antibiotic-resistant microorganisms are still present in retail poultry meat [65].

Beef lots and dairy farms have enacted relatively less intense and routine biosecurity measures compared to poultry and swine although there have been many improvements in understanding the benefits of space in feedlots for cattle biology and behavior (2.5 m^2^ to 3.0 m^2^ per animal) as well as straw bedding, group housing to improve wellness for veal calves and shade and enrichment in feedlots [66].

In dairy farms, one European study compared four housing systems and found that cows in the open-pack system with deep litter had lowest disease prevalence and longest productive herd life although all four systems had disadvantages and farms with even the same type of housing system can differ in housing quality [67]. Dairy managerial changes such as reducing the number of lactations per cow and raising replacement heifer calves off-site have reduced the presence of *Mycobacterium avium* subspecies *paratuberculosis* (Johne's disease) on some dairy farms. Although the impact of *M. avium* subspecies *paratuberculosis* may be decreased in dairy cattle, the incidence of this disease remains a concern for small ruminants, including goats and dairy goat farms have an increasing presence in the United States. The 2024 outbreak of HPAI H5N1 in dairy farms may be forcing new standards in terms of biosecurity for dairy cows, calves, animal caretakers, and other farm animals that come in contact with milk production systems (e.g., cats, rodents, birds).

Alongside managerial changes outlined above, NADC is continuing to better understand the etiopathogenesis, microbial and microbiome dynamics of food safety and mastitis pathogens as well as respiratory pathogens of cattle and swine. Also, the host immune responses, preventative measures, developing therapies and vaccines for these same pathogens are being studied. For example, NADC in collaboration with the University of Minnesota has a small herd of Holstein dairy cows with unselected genetics that date back to the year 1964; in other words, these Holstein cows lack modern genetic selection for high-milk-producing Holstein cows. They are used for mastitis infection studies along with genetic responses to infection and work has shown that these cattle have enhanced immune responses that successfully contain an *E. coli* intramammary infection [68] and have stronger immune responses than contemporary cows that have undergone genetic selection for milk production [24,69]. Also, NADC investigators have discovered that biocides can reduce the lesions of digital dermatitis (unpublished data), potato starch can reduce colonization of *Salmonella* spp. in pigs through gut immunity enhancement [70], vaccination of swine against *Salmonella* can limit spread and contamination [71], subunit formulations and deletion mutants of *M. avium* subspecies *paratuberculosis* are pro-apoptotic and thus good vaccine candidates [72,73], and polymorphisms in prion genotypes can affect virulence that could lead to identification of sheep and farmed deer resistant to Scrapie/Chronic wasting disease (CWD) [43].

## 3 Yet other reasons for persistence

Some diseases unfortunately may fall into the category of “not my problem”. Such diseases are either difficult to garner support to investigate or they do not fit a funding niche. Brucellosis, for example, is clearly a disease of humans worldwide but would be difficult to fund by a competitive granting agency such as the National Institute of Health (NIH) because Brucellosis is already identified as a “Program Disease” (designated by the USDA to have a “Program” to eliminate Brucellosis). Also, *Brucella canis* infection of dogs is of increased concern, but the NADC does not conduct research in dogs and there is, currently, not sufficient concern for this pathogen to cause disease in humans and receive funding from other sources. *E. coli* O157:H7 can cause severe disease in humans from consumption of undercooked beef but does not cause disease in cattle and thus, the cattle industry would likely be resistant to develop a vaccine against *E. coli* O157:H7 for cattle. And while vaccines are made against *Salmonella* spp. for poultry, there is less of an acceptable process for a *Salmonella* sp. vaccine for humans. Yet other diseases, such as *Listeria monocytogenes*, which causes meningitis and abortion in cattle and can also be a food pathogen, are not researched at NADC; nor is Chlamydophila spp. NADC research is bound by congressional mandates based on animal producer and stakeholder concerns. These diseases will continue to fall through the cracks until they become a more recognized problem such that industry or funding agencies will take notice.

These circumstances have led us to wonder what if funding and research were eliminated for these diseases? Regardless, of whether this came about from a lack of funding, policy change or outright ban, halting research would no doubt be devastating. It is hard to predict the specific impacts from such an ill-advised scenario, but most certainly infection rates and economic burdens would increase. Any progress or breakthroughs would halt, including vaccines, novel therapeutic treatments and other countermeasures. Innovation would be stifled, and disease eradication couldn't even be considered. Environmentally, with pathogens spreading at unchecked rates, bystander infections and interspecies infections would increase. Collectively, eliminating animal disease research will have a huge negative impact on One Health agendas focused on keeping animals and humans healthy.

## 4 Conclusions

NADC has faced numerous disease challenges during its 60-year existence and has used proven as well as innovative strategies to combat these conditions and make progress in reducing their impact [1]. Furthermore, the research has been agile enough to shift priorities when a rapid response is needed such as porcine reproductive and respiratory syndrome virus in swine, SARS-CoV-2 susceptibility in WTD (Table 1) and HPAI in swine and dairy cattle (Table 2). A selection of noteworthy accomplishments (Table 1) demonstrates the progress and there are many examples of how NADC quickly shifted priorities to address disease outbreaks (Table 2). The recent past and near future suggest that the national need for innovative animal health and food safety research at NADC, NBAF and elsewhere has never been greater. The increasing human population and natural resource utilization continues to put pressure on the environment, further reducing the animal habits. This leads to closer proximity of animals and resulting disease transmission opportunities to other animals and humans. Continued research in the target species of both livestock and wildlife remains a high priority to curb the damaging impact of animal disease in our nation.

## CRediT authorship contribution statement

**Mark R. Ackermann:** Writing – review & editing, Writing – original draft, Conceptualization. **John P. Bannantine:** Writing – review & editing, Writing – original draft, Conceptualization.

## Declaration of competing interest

None.

## Data Availability

Extensive research was conducted on the diseases and history that surrounds ARS' National Animal Disease Center. These data and sources are available from the corresponding author upon request.
